# Supplementary material for: Multipronged interventions to reduce surgical site infections: A multicenter implementation research protocol
Source: PLoS One. 2025 Mar 27;20(3):e0319645. doi: 10.1371/journal.pone.0319645 (PMC11949327; doi:10.1371/journal.pone.0319645)
Supplement: S1 File — (DOCX) [file pone.0319645.s001.docx]

Protocol R5-AM

| Complete Title | Impact of multipronged interventions targeting healthcare professionals in reducing surgical site infections and identifying the challenges in adoption of these interventions- An implementation research | |
| --- | --- | --- |
| Short Title | Multipronged interventions and surgical site infections | |
| Protocol Identification no. | RR/A5-AM/1.6 | |
| Principal Investigator | Dr. Rachna Rohilla | |
| Protocol Date and version | Version 1.6 | |
| Amendment 1 Date: 20 April 2023 | | Amendment 4 Date: 10 July 2023 |
| Amendment 2 Date: 20 May 2023 | | Amendment 5 Date: September 2023 |
| Amendment 3 Date: 13 June 2023 | | Amendment 6 Date: January 2024 |

**Confidential**

*This document contains confidential information. It is to be used by investigators, consultants, regulatory authorities or ethics committees.
The information is not to be disclosed to other parties without prior written permission*

*from ICMR and Principal Investigator except where required by applicable laws in India.*

**Contact Details**

**Principal Investigator:** Dr. Rachna Rohilla, Assistant Professor, Department of Pharmacology, AIIMS Bathinda

**Postal address:** B-308, Department of Pharmacology, Medical College Building, AIIMS Bathinda

**Telephone number:** 9876238583

**Email ID:** rachna.rohilla20@gmail.com

**Investigators**

| Central coordinating site: AIIMS Bathinda | Site PI: Dr. Rachna Rohilla, Assistant Professor, Pharmacology, AIIMS Bathinda  Site Co-PI: Dr. Mayank Gupta, Associate Professor, Anaesthesia, AIIMS Bathinda; Dr. Mahendra Pratap Singh, Additional Professor, General Surgery, AIIMS Bathinda |
| --- | --- |
| Six collaborating sites: | - Postgraduate Institute of Medical Education and Research, Chandigarh (PGIMER Chandigarh)   Site PI: Dr. Ashish Kumar Kakkar, Associate Professor, Pharmacology, PGIMER Chandigarh  Site Co-PI: Dr. Yashwant R Sakaray, Assistant Professor, General Surgery, PGIMER Chandigarh   - Postgraduate Institute of Medical Sciences (PGIMS Rohtak)   Site PI: Dr. Niti Mittal, Associate Professor, Pharmacology, PGIMS Rohtak  Site Co-PI: Dr. Sanjay Marwah, Professor, General Surgery, PGIMS Rohtak   - Dayanand Medical College, Ludhiana   Site PI: Dr. Sandeep Kaushal, Professor, Pharmacology, DMC Ludhiana  Site Co-PI: Dr. Jaspal Singh, Professor and Head of Department, General Surgery, DMC Ludhiana   - Government Medical College & Hospital, Sector-32, Chandigarh (GMCH-32)   Site PI: Dr. Robin Kaushik, Professor, General Surgery, GMCH-32, Chandigarh  Site Co-PI: Dr. Simrandeep Singh, Associate Professor, General Surgery, GMCH-32, Chandigarh   - Jawaharlal Nehru Medical College (JNMC), AMU, Uttar Pradesh   Site PI: Dr. Syed Shariq Naeem, Assistant Professor, Pharmacology, JNMC, AMU  Site Co-PI: Dr. Shahbaz Habib Faridi, Assistant Professor, Department of Surgery, JNMC, AMU  Dr. Mohammad Jesan Khan, Department of Orthopedics, JNMC, AMU   - Dr. Radhakrishnan Government Medical College, Hamirpur, Himachal Pradesh (H.P)   Site PI: Dr. Sanchit Chaudhary, Associate Professor, General Surgery, Dr. Radhakrishnan Government Medical College, Hamirpur  Site Co-PI: Dr. Shikhar Dogra, Assistant Professor, Orthopedics, Dr. Radhakrishnan Government Medical College, Hamirpur |
| Advisory Committee | - Dr. Anish TS, Professor, Department of Community Medicine, Government Medical College, Manjeri, Malappuram, Kerala. - Dr. Devi Vijay, Professor, Indian Institute of Management, Calcutta, Kolkata. |

Study Monitor:

Dr. Aparna Mukherjee

Scientist E and In-charge Clinical Studies and Trial Unit Division of Development Research Indian Council of Medical Research

Email ID: aparna.sinha.deb@icmr.gov.in

#

# Table of Contents

Table of Contents 4

Protocol Synopsis vii

1 Background Information and Rationale 1

1.1 Introduction and relevant literature 1

2 Study Objectives 3

2.1 Primary Objective (or AIM): 3

2.2 Secondary Objectives (or AIM) 3

3 Investigational plan 3

3.1 General Scheme of Study Design and Study Plan: 3

3.1.1 Phase-I (Formative research): 4

3.1.2 Phase-II (Co-development of a multipronged intervention package addressing the identified barriers and suitable for low resource settings by the stakeholders): 5

3.1.3 Phase-III (Deployment of the multipronged interventions): 8

3.1.4 Phase-IV (Impact assessment and results dissemination): 9

3.1.5 Supervision of the study implementation: 10

3.2 Allocation to Treatment Groups and Blinding (if applicable) 10

3.3 Study Duration, Enrollment and Number of Sites 10

3.3.1 Duration of Study Participation 10

3.3.2 Total Number of Study Sites/Total Number of Subjects Projected: 11

3.4 Study Population 12

3.4.1 Inclusion Criteria 12

3.4.2 Exclusion Criteria 12

3.5 Subject Completion/Withdrawal 12

3.5.1 Early Termination Study Visit 13

3.6 Description of study evaluations/measurements/assessments 13

3.7 Primary Endpoint 13

3.8 Secondary Endpoints 13

3.9 Statistical analysis plan 13

3.10 Analysis of Qualitative data 14

3.11 Sample Size and Power 14

4 STUDY ADMINISTRATION 15

4.1 Data collection and management 15

4.2 Confidentiality 15

4.3 Regulatory and Ethical Considerations 15

4.4 Recruitment Strategy 16

4.5 Informed Consent/Assent 16

5 PUBLICATION Plans 16

6 BUDGET (Per centre and TOTAL) 16

7 GANTT CHART 17

8 References 18

Appendix-I: Template example of the components of Multipronged interventions and CheckLIST to be checked for each patient 20

Appendix-II: who SURGICAL SITE INFECTION SURVEILLANCE POST-OPERATIVE DATA COLLECTION FORM 23

Appendix-IiI: DIAGNOSIS OF SURGICAL SITE INFECTION AS PER CDC 24

Appendix-IV: SURGICAL WOUND CLASSIFICATION AS PER CDC 25

Appendix-V: American society of anesthesiologist (ASA) physical status classification AS PER CDC 26

Abbreviations/Acronyms

| **Abbreviation** | **Expanded Form** |
| --- | --- |
| AHA | American Hospital Association |
| ASA | American Society of Anesthesiologists’ |
| APIC | Association for Professionals in Infection Control and Epidemiology |
| CDC | Centres for Disease Control and Prevention |
| DDD | Defined Daily Dose |
| DOT | Days of Therapy |
| HAI | Hospital Acquired Infections |
| ICU | Intensive Care Unit |
| IEC | Institutional Ethics Committee |
| IDSA | Infectious Disease Society of America |
| NHSN | National Healthcare Safety Network |
| OPD | Out Patient Department |
| POD | Post Operative Day |
| LMIC | Low- Middle Income Countries |
| SD | Standard Deviation |
| SHEA | Society for Health care Epidemiology of America |
| SPSS | Statistical package for the social sciences |
| SSIs | Surgical Site Infections |
| WHO | World Health Organization |

# Protocol Synopsis

| **Study Title** | Impact of multipronged interventions targeting healthcare professionals in reducing surgical site infections and identifying the challenges in adoption of these interventions. |
| --- | --- |
| **Type of study** | Implementation study |
| **Study Rationale** | While the global estimates of SSI vary from 0.5% to 15% depending on type of surgery; the low-middle-income countries including India have disproportionately higher rates of surgical site infections as ranging from 23 to 38% as compared to high-income countries, despite adjustment for the surgery and patient factors. SSI rate may be influenced by various factors like pre-operative care, timing of antimicrobial administration for surgical prophylaxis, patient factors, operation theatre environment, intra-operative conditions, type of surgery, post-operative care and hand hygiene. However, the implementation of these measures has not been standardised due to multiple reasons with some being lack of knowledge in surgeons, rigidity in changing the practice among surgeons and health care professionals despite availability of WHO recommendations.  Recent audits and point prevalence studies in LMICs including India confirm continued high occurrence of multiple doses of antimicrobial surgical prophylaxis for > 1day, which is likely to exacerbate antimicrobial resistance and contribute to added cost of therapy. 48.9% of the ICU patients have apparent inappropriateness in the choice of prophylactic antibiotic. Dual anaerobic coverage in 43.5%, dual gram-negative coverage in 9.7% and overlapping in both gram positive and gram-negative coverages in 4.4% patients. A recent meta-analysis by Cooper L et al (2020) showed that administration of single-dose and short-duration surgical antimicrobial prophylaxis in caesarean section was associated with lower risk of SSI. However, the effect of adoption of optimal surgical prophylaxis remains under-evaluated in other type of surgeries including general surgery. With this background, we plan to develop and assess the effect of multipronged interventions for prevention of surgical site infections in surgical patients. |
| **Study Objective(s)** | **Primary**  To evaluate the effect of multipronged interventions on rate of surgical site infections in clean, clean-contaminated, contaminated surgeries.  **Secondary**   1. To identify the system and behavioral factors contributing to surgical site infections 2. To co-develop multipronged interventions which can be applied in hospital settings 3. To evaluate the impact of multipronged interventions on inpatient mortality and length of hospital stay 4. To evaluate the number of deaths prevented by adoption of multipronged interventions 5. To evaluate the number of ICU admissions averted and number of hospital readmissions averted within 30 days after discharge by adoption of multipronged interventions 6. To evaluate the effect of multipronged interventions on the antimicrobial consumption indicator Days of therapy (DOT). 7. To evaluate the percentage of patients receiving the correct surgical antimicrobial prophylaxis. |
| **Intervention** | Multipronged Interventions (to be co-developed by stakeholders) |
| **Indication** | Prevention of surgical site infections |
| **Study Design** | Mixed methods- Co-development of intervention (Mixed method) followed by Quasi-experimental, pretest-posttest design (Quantitative) and in-depth-interviews of healthcare professionals (Qualitative) |
| **Subject Population**  **key criteria for Inclusion and Exclusion:** | **Inclusion Criteria**   1. Surgical adult patients undergoing elective or emergency procedure (open or laparoscopic) under general surgery, neurosurgery, orthopedics, plastic surgery, urology, pediatric surgery. 2. Aged upto 65 years of age.   **Exclusion Criteria**   1. Pregnant or lactating females 2. Immunocompromised patients including uncontrolled diabetic patients, cancer patients, on immunosuppressive therapy. 3. HIV/HBV/HCV infection 4. Patients with prolonged hospital stay >1 week and received multiple antimicrobials before the planned procedure. 5. Patients with dirty wounds 6. Pre-existing active infection at surgical site 7. Minor procedure performed under local anesthesia 8. Therapeutic hypothermia needed during intra-operative or post-operative period |
| **Number of Participants** | The three types of surgeries (clean, clean-contaminated and contaminated) will be separately powered, based on different baseline SSI rates. The sample sizes were based on 90% power, a 5% two-sided significance level and 15% loss to follow up or death before reaching the primary endpoint at 30-days. For the clean surgeries, anticipating the baseline average SSI rate of 8%, a 3% absolute reduction in SSI to 5% will be taken as clinically significant and would require 3260 patients in total. For the clean-contaminated surgeries, anticipating the baseline average SSI rate of 12%, a 4% absolute reduction in SSI to 8% will be taken as clinically significant and would require 2700 patients in total. For the contaminated surgeries, anticipating higher baseline SSI rate of 30%, a 10% absolute reduction in SSI to 20% will be taken as clinically significant and would require 900 patients in total. |
| **Study Duration** | Each subject’s participation will last for 30 days or 90 days if implant in situ. |
| **Study Phases** | The plan is to adopt a four- phased strategy, each addressing specific research question. Phase-I will involve formative research and practice analysis. Phase-II will involve co-development of the multipronged interventions. Phase-III will involve deployment of the multipronged interventions and process evaluation. Phase-IV will involve impact assessment and dissemination of results. |
| **Primary endpoint** | The rate of surgical site infection till 30 days after surgery (90 days if implant) using CDC definition of SSI in pre-intervention versus post-intervention phase for clean, clean-contaminated and contaminated surgeries |
| **Secondary endpoints** | 1. The number of ICU admissions averted and number of readmissions averted in clean, clean-contaminated and contaminated surgeries by adoption of multipronged interventions 2. The length of index hospital admission in pre-intervention and intervention phase for clean, clean-contaminated and contaminated surgeries. 3. The antimicrobial consumption indicator, Days of therapy (DOT) in the pre-intervention and intervention phase for clean, clean-contaminated and contaminated surgeries. 4. The percentage of patients receiving single dose of antimicrobial prophylaxis in the pre-intervention and intervention phase for clean, clean-contaminated and contaminated surgeries 5. The percentage of patients receiving prolonged antimicrobial prophylaxis (>24 hours duration) in the pre-intervention and intervention phase for clean, clean-contaminated and contaminated surgeries 6. The percentage of irrational combinations of drugs (double gram positive, double gram negative or double anaerobic coverage) for surgical antimicrobial prophylaxis in the pre-intervention and intervention phase for clean, clean-contaminated and contaminated surgeries 7. The number of deaths prevented in clean, clean-contaminated and contaminated surgeries by adoption of multipronged interventions 8. Acceptance of multimodal interventions and challenges in their implementation by healthcare professionals. |
| **Statistical And Analytic Plan** | The data collected in the antimicrobial audit form will be analysed after entry into the excel form. The antimicrobial consumption analysis will be done using Microsoft excel. The descriptive data analysis will be done using Statistical package for the social sciences (SPSS) Version 23. The rate of surgical site infection till 30 days after surgery (90 days if implant) will be expressed as events per person-time, separately for clean, clean-contaminated, contaminated surgeries. All secondary endpoints will be expressed as mean, SD (if the variable is quantitative) or as frequency, percentage (if the variable is categorical). The rates of primary outcome and secondary outcomes measured at the study centres in post-intervention will be compared with the pre-intervention/baseline data to assess the impact of the intervention. Chi-square test, independent sample t test or Mann-Whitney U test will be used for the statistical comparisons.  Plots and pivot tables will be generated using statistical software or excel as appropriate. The analysis will be conducted separately for clean, clean-contaminated and for contaminated surgeries. A sub-group analysis will also be conducted as per the level of fidelity.  Kaplan Meier curves will be plotted to demonstrate the incidence rates of surgical site infections. The difference in the rate of surgical site infections between the nature of surgeries (clean, clean-contaminated, contaminated surgeries), or any other exposure categories will be statistically tested using the log-rank test. A Cox proportional hazard model will be constructed to identify the predictors of surgical site infections. |
| **Safety Evaluations** | The study does not involve any additional risk to baseline the risk of surgery. So, we will request the Ethics Committee (EC) for waiver of the informed consent. |
| **Data And Safety Monitoring Plan** | The study involves low-minimal risk. The confidentiality and anonymity of each patient and their data will be strictly maintained before, during the study and while publication of the study results. |

# Background Information and Rationale

## Introduction and relevant literature

Surgical site infections (SSIs) are defined as the superficial site involving skin and subcutaneous tissue of the incision site within 30 days or deep incisional site infections involving deep soft tissues of the incision (fascial and muscle layers) within 30 or 90 days following the operative procedure with the patient having at least one of the following: (a) purulent discharge from the incision, (b) organism(s) detected from the aseptically obtained wound specimen, (c) evidence of infection or abscess (in case of deep SSI) by clinical examination or at imaging, or incision opened spontaneously or deliberately opened by the clinician/surgeon; and patient had at least one of the following signs and symptoms: localized pain or tenderness, localised swelling, redness, heat at the wound site or systemic fever (>38°C) as per Centres for Disease Control and Prevention (CDC).^1^

The World Health Organization (WHO) mentions SSI as the most common hospital acquired infections (HAI) among surgical patients in low-middle income countries (LMICs) affecting around one third of the patients undergoing surgical procedures.^2^ While the global estimates vary from 0.5% to 15% depending on type of surgery; the LMICs including India have disproportionately higher rates of surgical site infections as ranging from 23 to 38% as compared to high-income countries,^3^ despite adjustment for the surgery and patient factors.^4^ SSIs lead to higher antibiotic consumption, increased medical costs, prolonged hospital stay and recovery time, poor wound healing, risk of wound breakdown and hernia, poor clinical outcomes, psychological challenges and increased mortality. The patients who develop SSI are 60% more likely to be admitted to ICU and have twice the mortality rate than non-infected patients.^3^ It is noteworthy that around 40-60% of SSIs are preventable by use of infection control practices. The Society for Health care Epidemiology of America (SHEA) in collaboration with Infectious Disease Society of America (IDSA), the American Hospital Association (AHA), the Association for Professionals in Infection Control and Epidemiology (APIC) published “Strategies to prevent surgical site infections in acute care hospital” in 2008 and updated in 2016 and WHO provides the guidance to prevent the surgical site infections.^2,5,6^

Despite these guidance documents in place, SSIs remains a substantial cause of morbidity, mortality and economic burden in hospitalized surgical patients especially in LMICs. Recent studies from India showed a comparatively lower yet substantial incidence of SSI of about ~12.5 % in general surgical patients with most common pathogen associated with SSI being beta-haemolytic *Streptococci* and *Staphylococcus aureus*.^7^ The study found that the prevalence of SSI was higher (17.7%) in emergency surgeries as compared to 12.5% in elective surgeries probably requiring judicious and timely use of surgical prophylaxis as per recommendations, extra pre-operative and intra-operative care to lower overall SSI incidence rates.^7^ Similar rates were found in a recent large pragmatic multicentric randomized controlled study in LMICs with overall SSI rate of 22% (15.5% for clean-contaminated and 30% for contaminated or dirty surgeries).^8^ A prospective study conducted recently in PGIMS Rohtak, India found incidence of SSI to be ~8% for clean and ~10% for clean-contaminated surgeries.^9^

SSI rate may be influenced by various factors like pre-operative care, agent and timing of antimicrobial administration for surgical prophylaxis, patient factors, operation theatre environment, intra-operative conditions, type of surgery, post-operative care and hand hygiene.^10^ However, **the implementation of these measures has not been standardised due to multiple reasons** with some being lack of knowledge in surgeons, rigidity in changing the practice among surgeons and health care professionals, inconsistency in interpretation of evidence and WHO recommendations. The WHO describes compliance with current guidelines for antimicrobial surgical prophylaxis, surgical prophylaxis within 60 minutes prior to surgery and surgical prophylaxis stopped within 24 hours after surgery as major process measures in reducing the surgical site infections.^11^ In addition to appropriate use of antimicrobial prophylaxis, other measures like maintaining operation theatre (OT) cleanliness, hand hygiene, aseptic precautions, sterile environment in OT, restricting the opening of OT door are other important measures in limiting the SSIs^12,13^. A recent quality improvement study in cesarian section patients in India showed that quality improvement measures like following standard antimicrobial prophylaxis guidelines, decreasing OT traffic, reduced door openings training of OT staff, surgical safety checklist, and biomedical waste management led to reduction in SSI rate from 30% to 5% over a period of 6 months^14^.

Recent audits and point prevalence studies in LMICs including India confirm continued high occurrence of multiple doses of antimicrobial surgical prophylaxis for > 1day, which is likely to exacerbate antimicrobial resistance and contribute to added cost of therapy.^15,16^ Another study done to see antibiotic prescription practices for surgical prophylaxis in Bangalore, India found that 48.9% of the ICU patients had apparent inappropriateness in the choice of prophylactic antibiotic and only 3.2 % were in accordance with the IDSA guidelines. Dual anaerobic coverage was present in 43.5%, dual gram negative coverage was present in 9.7% and overlapping in both gram positive and gram negative coverages was present in 4.4% of the total 1012 patient ICU days, exposing the patients to higher WHO defined daily doses (DDD) of antibiotics.^17^ A recent meta-analysis by Cooper L et al (2020) showed that education to improve appropriate antibiotic prophylaxis is associated with reduction of SSIs in LMICs.^18^ Even though the sizes of absolute effect were small (risk ratio of 0.77 and 0.89) and not statistically significant, administration of single-dose and short-duration surgical antimicrobial prophylaxis in Caesarean section was associated with lower risk of SSI.^18^

To the best of our knowledge, efficacy of optimal surgical prophylaxis has not been strategically evaluated in other type of surgeries including general surgery. There is evidence in the literature that for surveillance to improve outcomes it needs to be paired with effective audit and feedback mechanism to frontline workers, so that they can change their behaviour in light with the quantitative data of audit and feedback.^13^ However, this alone has not led to significant reduction in the rate of SSIs. Hence, there is a need to co-develop feasible and scalable multipronged interventions, targeting healthcare professionals, system level challenges and contextualised to the logistic constraints of LMICs for prevention of surgical site infections.

# Study Objectives

## Primary Objective (or AIM):

To evaluate the effect of multipronged interventions on rate of surgical site infections in clean, clean-contaminated, contaminated surgeries (Refer to Appendix III for definitions). (Multipronged intervention components mentioned in section 3.6)

## Secondary Objectives (or AIM)

1. To identify the system and health care professional behavioral factors contributing to surgical site infections
2. To co-develop multipronged interventions which can be applied in hospital settings in LMICs
3. To evaluate the impact of multipronged interventions on in-patient mortality and length of hospital stay
4. To evaluate the number of deaths prevented by adoption of multipronged interventions
5. To evaluate the number of ICU admissions averted and number of hospital readmissions averted within 30 days after discharge by adoption of multipronged interventions
6. To evaluate the effect of multipronged interventions on the antimicrobial consumption indicators Days of therapy (DOT).

[Days of therapy is defined as an aggregate sum of days for which any amount of a specific antimicrobial agent was administered to individual patient^13^]

1. To evaluate the percentage of patients receiving the correct surgical antimicrobial prophylaxis.
2. To evaluate the acceptance of these multipronged interventions and challenges in their implementation by health care professionals using in-depth interviews and a qualitative feedback assessment form.

# Investigational plan

## General Scheme of Study Design and Study Plan:

Quasi-experiment (pre-post design) will be adopted.

The plan is to adopt a four- phased strategy using mixed-method approach, each addressing specific research question (Figure-1).

**Figure-1: Phase-wise research strategy, objectives and activities planned for each phase of the study.**

### Phase-I (Formative research):

Mixed-method design will be adopted.

Phase-I will involve the scoping review to assess the knowledge, attitude and practice (KAP) gap from the literature in LMIC setting. After scoping review, we will conduct a mixed-method analysis in the form of a sequential exploratory design, cross-sectional analysis of administrative data (QUAN) followed by IDIs of surgeons (QUAL).

The quantitative analysis involves the baseline data collection on the burden of SSI and evaluation for surgical practices among healthcare professionals using a cross-sectional analysis (quantitative part). The risk factors which might contribute to the increased risk of SSI will be identified in the settings involved in the study. The qualitative practice assessment form will be formulated using the semi-structured questionnaire to be filled by the surgeons on what practice they follow for various type of surgeries (elective and emergency surgeries) in their setting and if there are any challenges in adoption of the already published guidelines (global guidelines for prevention of surgical site infections by WHO^2^, second edition 2018 and Clinical practice guidelines for antimicrobial prophylaxis in surgery^5^). A semi-structured format allows us to remain open to exploratory findings during the course of interviews.

The qualitative component of this phase of analysis involves In-depth interviews (IDI) with the surgeons will be conducted to explore their perspectives and perceived barriers on implementation of SSI prevention strategies in their respective settings. The number of IDIs will range from 10-15 or until saturation of data is achieved. In addition, the IDI will be conducted with nurses to understand what surgical pre-operative, operative and post-operative measures they follow in routine surgeries in operation theatre and what are the challenges in adoption of already published surgical site infection prevention guidelines as mentioned above?

The baseline data collection will be done by the trained research associate/ICN nurse for the patients undergoing surgeries as per inclusion/exclusion criteria and the baseline SSI rate in the setting will be calculated from the baseline data collected. The baseline data collection will be done using the standard case record form (CRF) designed for the study which will include collection of data on (but not limited to) parameters like type of surgery, American Society of Anesthesiologists (ASA) physical status score (refer to Annexure-V), timing and antimicrobial agent for surgical prophylaxis, duration of surgery, need for redosing of antimicrobial prophylaxis, and other defined parameters. In addition, the observation of practices of the care delivery team and observation of interactions among care team and patients will be done at this stage. This qualitative assessment will allow the research team to observe points that practitioners may not have reflected on or recollect during interviews. The baseline data collection phase will last for 6 months. No intervention or practice change will be applied in this phase.

**Outcome of Phase-I:** Expected outcome of Phase-I will be to identify the knowledge gaps in the practice, barriers and challenges in adoption of the surgical prophylaxis prevention guidance. The baseline data collection will be done in this phase (pre-intervention data) on the baseline SSIs rates and practice followed in the setting. These findings will be helpful in co-development of the multipronged interventions during Phase-II.

### Phase-II (Co-development of a multipronged intervention package addressing the identified barriers and suitable for low resource settings by the stakeholders):

The multipronged interventions will be co-developed by the stakeholders based on the findings of scoping/literature review, baseline assessment, and available guidance. The stakeholders will involve the head of the departments of surgery, hospital administration (medical superintendent of the site), the investigators (PI and Co-PI) from respective sites involved. All the stakeholders will identify the practice gap in their setting from baseline data assessment. The most relevant, feasible and impactful interventions from observed clinical practices during baseline data collection phase, published guidance documents, scoping/literature review which are feasible as well as crucial in reducing the surgical site infections will be identified. [**Template example provided in Appendix-I**].

Series of discussions with all the stakeholders will be conducted to discuss the identified components of multipronged intervention. Any challenges foreseen in acceptability of the interventions will be discussed with the stakeholders before finalizing the components of the multipronged interventions. These interventions will then be mapped and all the stakeholders will reach on the consensus on which interventions to choose.

After finalizing the multipronged interventions among the stakeholders, the review will be done by the Project Advisory Group (PAG). The PAG will involve the senior academicians, external expert in the implementation research and PIs from the sites. The final multipronged interventions will be covering various core domains: system, structural and behavioral domains which will be implemented in the Phase-III as an intervention package.

After co-development, the standardization and monitoring plan of these multipronged interventions will be done as mentioned below (Figure 2)

**Figure 2: Development and standardization process of multipronged interventions**

**Intervention Standardization:** The steps of intervention standardization will include defining the type of standardization for each component and defining the limits of the standardization for each component^19^. The type and limits of the standardization will be set after consensus with the stakeholders and the project advisory group.

1. Type of standardization for each component and step can be classified into “mandatory” or “optional” or “prohibited”. “Mandatory” step must be performed under all circumstances and if not performed will be considered as protocol deviation; opposite is true for “Prohibited”. “Optional” step may or may not be performed at discretion of the participating surgeon.
2. Limits of standardization for each component can be set as “exactly” or “with boundaries” or “without boundaries (flexible)” based on the flexibility allowed for adoption by the health care professional. If mentioned as “with boundaries”, the component or step can be followed as per the amount of flexibility mentioned for each component of intervention. (Table -2)

**Intervention Monitoring:** The levels of fidelity will be defined and noted as per the levels mentioned in Table-1 after discussion with all the stakeholders. The discrepancy among the stakeholders will be resolved after discussion among each other for reaching to the final consensus. Based on the multipronged interventions, a checklist and standard data collection form will be developed. The check list developed will be used for assessing the level of fidelity and compliance to the multipronged interventions for each patient as shown is Appendix-I.

**Table-1**: Levels and type of intervention fidelity to be described for each component of the multipronged intervention^19^

| **Level of fidelity** | **Type and description** |
| --- | --- |
| Protocol deviation from intended intervention | Did not receive any intervention or received alternative intervention not being evaluated in the study |
| Deviation from component(s) of the intended intervention | Did not receive the component of the intervention or component of intervention delivered partially or component of intervention delivered in a different way than what is mentioned in the study protocol. |

The set of selected multipronged intervention package co-developed and standardized by all the stakeholders will be implemented among health care professionals.

**Outcome of Phase-II:** The expected outcome of Phase-II is to co-develop feasible and acceptable multipronged intervention package that can be implemented in the low-resource setting to prevent surgical site infections.

### Phase-III (Deployment of the multipronged interventions):

Phase-III will involve deployment of the multipronged interventions and process evaluation.

**Deployment of multipronged interventions:** Multipronged interventions developed in phase-II will be implemented in Phase-III using the following strategies: training of healthcare professionals, identification of Surgical site infection prevention (SSIP)/Stewardship champions, review of implementation by the project advisory committee.

**A. Structured training curriculum and training manual**: The structured training modules will be developed as per the multipronged interventions co-developed in Phase-II. The training modules will be contextualized to the practice change needs and the training sessions will be conducted using interactive sessions. The assessment will be applied pre and post-training among the health care professionals undergoing training to assess the practice and knowledge change adopted from the training modules. The assessment will also indicate the need for re-training.

**B. Training**: The project steering group (comprising of multidisciplinary team of PIs from the sites, antimicrobial stewardship experts) will impart comprehensive training of the relevant health care professionals including the operation theatre nursing staff involved in surgery and patient care, the residents, anesthesiologists’ and the surgeons using the structured training modules and curriculum using 4-5 interactive sessions, didactic lectures for adoption of the multimodal interventions spanned over a period of two months. The health care professions undergoing training will be assessed for practice and knowledge change using pre-developed assessment module. The score of >80% in the training assessment will be considered adequate. If score of <80%, then either re-training session will be conducted or not included in the study.

**C. Identification of SSIP/Stewardship Champions**: The “SSIP champions” will be identified for each setting by the site PI/Co-PI (could be ICN nurse/resident/surgeon who have leadership qualities and actively working in the surgery department). These SSIP champions will ensure the adoption of the multimodal interventions in their setting and will also assess the need of interim training or re-training in their respective sites. If the compliance to the multipronged interventions is not there at a particular site as assessed by the checklist of each patient, then re-training will be schedule. If non-compliance is persistent, then it will be communicated to the project advisory group. The process evaluation will be done by project advisory group and IDI will be conducted to understand the hurdles and challenges in adoption of these interventions and explained below.

**Process evaluation**: The compliance to the multipronged interventions as assessed by the SSIP champions will be evaluated by the project advisory committee. The SSIP champions will also evaluate whether the structural and system changes are in-place at respective sites and behavioural changes are adopted by the health care professionals. If there is non-compliance and is persistent, then the stakeholders will re-assess the acceptance of the co-developed multipronged interventions. In-depth interviews with the health care professionals will follow to understand the hurdles and challenges in accepting these interventions and troubleshooting iteratively. The multipronged interventions will be either adapted as per the challenges evolving or co-developed as per the site need or if only retraining will suffice, then re-training will be planned. The monitoring of the compliance to the multipronged interventions at each site using the checklist will be done by SSIP champions and reported to the project advisory group quarterly.

The intervention will continue with the help of SSI Champions and after the study is over also. The intervention and result assessment quarterly will go hand in hand and the impact assessment will follow.

### Phase-IV (Impact assessment and results dissemination):

Phase-IV involve the post-intervention phase for assessing the impact of adoption of the multipronged interventions in prevention of SSIs and the dissemination phase.

**Post-intervention Phase**:

This phase will involve the data collection on the same parameters as in pre-intervention phase using the CRF and check the implementation of multipronged intervention packages using the checklist developed based on the multipronged interventions. The SSIP champions will evaluate whether these multipronged interventions are adopted adequately in their respective setting. Intermittent weekly reminders like e-posters, WhatsApp messages will be given to health care professionals for adoption of the multimodal interventions which will act as re-enforcement reminders. Interim training sessions will be planned after every 6 months (in case new staff is recruited or compliance to interventions reduces).

In-depth interviews will be conducted with key stakeholders (surgeons, health care professionals and nursing officials involved) to evaluate their perception of impact and feasibility of interventions at 6 months post-intervention phase and suggestions to improve the multipronged interventions.

**Dissemination Phase:**

The report of the project will be presented to the Head of the Institute of the respective institute and project advisory group. The results will be published in the indexed journal as mentioned in Section 5 “Publication Plans” below.

**Figure-3**: Study plan and duration of each phase of the study

### Supervision of the study implementation:

The project advisory group (PAG) will maintain the audit trail of all the activities done in their respective sites. Periodic meetings will be planned (physical or virtual with all the stakeholders and they will be appraised of the project activities. The plan is to have at least 5 physical meetings with all stakeholders (one before starting the study, three during study and one after study completion). The implementation plan will be continuously evolved with the inputs for the project advisory group and external experts in implementation research.

## Allocation to Treatment Groups and Blinding (if applicable)

Not applicable

## Study Duration, Enrollment and Number of Sites

### Duration of Study Participation

The study participation will last for 30-days or 90 days if implant placement during surgery. Each subject will be included in the study on pre-operative day-1 and followed till discharge. After discharge, the patient will be reviewed in the OPD or followed up telephonically till 30-days of the surgery or 90 days if implant placed (where the day of surgery will be taken as POD-0) for any evidence of surgical site infection. Any re-admission in-between the discharge and 30-days will be noted along with the reason of re-admission.

### Total Number of Study Sites/Total Number of Subjects Projected:

Seven sites/~1000-1500 patient data per site projected.

Central coordinating site: All India Institute of Medical Sciences (AIIMS Bathinda)

- PI: Dr. Rachna Rohilla, Assistant Professor, Pharmacology, AIIMS Bathinda
- Co-PI: Dr. Mayank Gupta, Associate Professor, Anaesthesia, AIIMS Bathinda; Dr. Mahendra Pratap Singh, Additional Professor, General Surgery, AIIMS Bathinda

Other participating sites:

- Postgraduate Institute of Medical Education and Research, Chandigarh (PGIMER Chandigarh)

Site PI: Dr. Ashish Kumar Kakkar, Associate Professor, Pharmacology, PGIMER Chandigarh

Site Co-PI: Dr. Yashwant R Sakaray, Assistant Professor, General Surgery, PGIMER Chandigarh

- Postgraduate Institute of Medical Sciences (PGIMS Rohtak)

Site PI: Dr. Niti Mittal, Associate Professor, Pharmacology, PGIMS Rohtak

Site Co-PI: Dr. Sanjay Marwah, Professor, General Surgery, PGIMS Rohtak

- Dayanand Medical College, Ludhiana

Site PI: Dr. Sandeep Kaushal, Professor, Pharmacology, DMC Ludhiana

Site Co-PI: Dr. Jaspal Singh, Professor and Head of Department, General Surgery, DMC Ludhiana

- Government Medical College & Hospital, Sector-32, Chandigarh (GMCH-32)

Site PI: Dr. Robin Kaushik, Professor, General Surgery, GMCH-32, Chandigarh

Site Co-PI: Dr. Simrandeep Singh, Associate Professor, General Surgery, GMCH-32, Chandigarh

- Jawaharlal Nehru Medical College (JNMC), AMU, Uttar Pradesh

Site PI: Dr. Syed Shariq Naeem, Assistant Professor, Pharmacology, JNMC, AMU

Site Co-PI: Dr. Shahbaz Habib Faridi, Assistant Professor, Department of Surgery, JNMC, AMU

Dr. Mohammad Jesan Khan, Department of Orthopedics, JNMC, AMU

- Dr. Radhakrishnan Government Medical College, Hamirpur, Himachal Pradesh (H.P)

Site PI: Dr. Sanchit Chaudhary, Associate Professor, General Surgery, Dr. Radhakrishnan Government Medical College, Hamirpur

Site Co-PI: Dr. Shikhar Dogra, Assistant Professor, Orthopedics, Dr. Radhakrishnan Government Medical College, Hamirpur

- Dr. Devi Vijay, Professor, Indian Institute of Management, Calcutta
- Dr. Anish TS, Professor, Department of Community Medicine, Government Medical College, Manjeri, Malappuram, Kerala.

## Study Population

### Inclusion Criteria

1. Surgical patients undergoing elective or emergency procedure (open or laparoscopic) under general surgery, neurosurgery, orthopedics, pediatric surgery, plastic surgery, urology.

### Exclusion Criteria

1. Pregnant or lactating females
2. Immunocompromised patients including uncontrolled diabetic patients, cancer patients, on immunosuppressive therapy.
3. HIV/HBV/HCV infection
4. Patients with prolonged hospital stay >1 week and received multiple antimicrobials before the planned procedure.
5. Patients with dirty wounds
6. Pre-existing active infection at surgical site
7. Minor procedure performed under local anesthesia
8. Therapeutic hypothermia needed during intra-operative or post-operative period
9. Patients with American Society of Anesthesiologist (ASA) score of 6 (declared brain dead whose organs are being removed for donor purposes) (For ASA score refer to Annexure-V)

## Subject Completion/Withdrawal

The patients who take leave from hospital against medical advice (LAMA) will be contacted for follow-up at 30-days for the outcome.

If follow-up is not possible at 30 days after surgery, patients will be followed up as soon after this as possible.

### Early Termination Study Visit

The site(s) which is/are not complying with the study procedure and the surgeons not following the multimodal interventions as required, will be terminated from the study. No further data collection will be done for that site.

## Description of study evaluations/measurements/assessments

## Primary Endpoint

The rate of surgical site infection till 30 days after surgery (90 days if implant) using CDC definition of SSI in pre-intervention versus post-intervention phase for clean, clean-contaminated and contaminated surgeries (Annexure-II, III).

## Secondary Endpoints

1. The number of ICU admissions averted and number of readmissions averted in clean, clean-contaminated and contaminated surgeries by adoption of multipronged interventions
2. The length of index hospital admission in pre-intervention and intervention phase for clean, clean-contaminated and contaminated surgeries.
3. The antimicrobial consumption indicators (Days of therapy) in the pre-intervention and intervention phase for clean, clean-contaminated and contaminated surgeries.
4. The percentage of patients receiving single dose of antimicrobial prophylaxis in the pre-intervention and intervention phase for clean, clean-contaminated and contaminated surgeries
5. The percentage of patients receiving prolonged antimicrobial prophylaxis (>24 hours duration) in the pre-intervention and intervention phase for clean, clean-contaminated and contaminated surgeries
6. The percentage of irrational combinations of drugs (double gram positive, double gram negative or double anaerobic coverage) for surgical antimicrobial prophylaxis in the pre-intervention and intervention phase for clean, clean-contaminated and contaminated surgeries
7. The number of deaths prevented in clean, clean-contaminated and contaminated surgeries by adoption of multipronged interventions
8. Acceptance of these multipronged interventions and challenges in their implementation by healthcare professionals.

## Statistical analysis plan

The data collected in the antimicrobial audit form will be analysed after entry into the excel form. The antimicrobial consumption analysis will be done using Microsoft excel. The descriptive data analysis will be done using Statistical package for the social sciences (SPSS) Version 23. The rate of surgical site infection till 30 days after surgery (90 days if implant) will be expressed as events per person-time, separately for clean, clean-contaminated, contaminated surgeries. All secondary endpoints will be expressed as mean, standard deviation (if the variable is quantitative) or as frequency, percentage (if the variable is categorical). The rates of primary outcome and secondary outcomes measured at the study centres in post-intervention will be compared with the pre-intervention/baseline data to assess the impact of the intervention. Chi-square test, independent sample t test or Mann-Whitney U test will be used for the statistical comparisons.

Plots and pivot tables will be generated using statistical software or excel as appropriate. The analysis will be conducted separately for clean, clean-contaminated and for contaminated surgeries. A sub-group analysis will also be conducted as per the level of fidelity.

Kaplan Meier curves will be plotted to demonstrate the incidence rates of surgical site infections. The difference in the rate of surgical site infections between the nature of surgeries (clean, clean-contaminated, contaminated surgeries), or any other exposure categories will be statistically tested using the log-rank test. A Cox proportional hazard model will be constructed to identify the predictors of surgical site infections.

## Analysis of Qualitative data

## We will have qualitative data in two phases of our study. IDIs will be conducted in phase 1 (formative study) as the design of the same is a mixed-method, sequential exploratory design. IDIs will also be conducted with key stakeholders (surgeons, health care professionals and nursing officials involved) to evaluate their perception of the impact and feasibility of interventions at 6 months post-intervention phase and suggestions to improve the multipronged interventions (Phase 4). Thematic analysis will be the analytical framework used for the qualitative analysis.

All interviews will be transcribed in English. The PI and Co-PIs will read through the responses in the qualitative practice assessment, observation notes and interview transcripts, creating memos where necessary. All responses will be thematically coded to facilitate the identification and analysis of patterns or themes in the data set ^21,22^. Themes will be generated from the initial codes. Themes will be reviewed, named and documented. Multiple thematic codes will be shared with all members of the research team to guide the development of the multipronged interventions.

## Sample Size and Power

The three strata based on the type of the surgery (clean, clean-contaminated and contaminated) will be separately powered, based on different baseline SSI rates extracted from Indian study showing prevalence of SSIs in general surgery patients.^7,8^ The sample sizes were based on 90% power, a 5% two-sided significance level and 15% loss to follow up or death before reaching the primary endpoint at 30-days. For the clean surgeries, anticipating the baseline average SSI rate of 8%, a 3% absolute reduction in SSI to 5% will be taken as clinically significant and would require 3260 patients in total (1630 patients in pre-intervention and intervention each). For the clean-contaminated surgeries, anticipating the baseline average SSI rate of 12%, a 4% absolute reduction in SSI to 8% will be taken as clinically significant (i.e relative risk of 0.67) and would require 2700 patients in total (1350 in pre-intervention and intervention each). For the contaminated surgeries, anticipating higher baseline SSI rate of 30%, a 10% absolute reduction in SSI to 20% will be taken as clinically significant and would require 900 patients in total (450 in pre-intervention and intervention each).

# STUDY ADMINISTRATION

## Data collection and management

Patients planned for general surgical procedures (elective or emergency) will be screened for inclusion in the study based on inclusion and exclusion criteria.

The data collection during baseline, post-intervention phase for the surgical patients undergoing elective or emergency surgery will be done by the Infection control nurse and research fellow in the standardized data collection form including but not limited to the data on timing of antimicrobial prophylaxis given, time of start of surgery, vitals, re-dosing of antimicrobial prophylaxis, time of stoppage of surgery, type of surgery (name of surgery; clean/clean-contaminated/contaminated/dirty), aseptic precautions taken or not, information on drains in situ, post-operative care, details of post-operative antimicrobials. The patient will be followed up regularly till discharge and data on SSI, antimicrobials, clinical outcome will be recorded. The patient will be called in-person for follow-up to ODP at 30-day to know the clinical status and outcome of the patient. Any unplanned visit to OPD or emergency will be noted with the reason of the visit.

If follow-up is not possible at 30 days after surgery, patients will be followed up as soon after this as possible or telephonically. If patient developed SSI before postoperative day 30, they will still be reviewed at 30 days after surgery to record secondary outcomes.

In intervention phase, in addition the checklist of multipronged interventions will be checked for implementation assurance along with the data collection form.

## Confidentiality

The anonymized data will be collected in the standard data collection form. The confidentiality of the data will be maintained during, after the study and during publication of the results.

## Regulatory and Ethical Considerations

The study will follow all the principles laid down by Declaration of Helsinki.

The study will commence after approval from the Institutional Ethics Committee from the Central coordinating site (AIIMS Bathinda) and each participating site.

## Recruitment Strategy

The patient planned for elective surgery will be screened as per inclusion-exclusion criteria a day prior to the surgery. For emergency surgeries, the screening will be done as earliest possible before planned emergency surgery (general surgery or neurosurgery).

## Informed Consent/Assent

Since, the multimodal interventions are based on already existing guidelines by WHO and IDSA, there is no involvement of any new intervention/drug, the study is low-minimal risk. Thus, we will request the Ethics Committee (EC) for waiver of the informed consent for this study.

# PUBLICATION Plans

We plan to publish the protocol and the manuscript in the PubMed indexed journal preferably “Journal of Antimicrobial Chemotherapy” or “International Journal of Antimicrobial Agents”.

# TOTAL BUDGET (TOTAL for all 7 centers)

| **S.NO** | **Particulars cumulative of all 7 centers** | **Year 1** | **Year 2** | **Year 3** | | **Total 3 years** |
| --- | --- | --- | --- | --- | --- | --- |
|  | **Staff/ Manpower** for the duration of | 10 months | 12 months | 10 months | | 32 months |
|  | 1. Project Technical Support-III (**5% increment in 3^rd^ year**)   @ 28,000 + 9% HRA = ~30,520 INR per month per centre (*4 centres)  @ 28,000 + 18% HRA = ~33,040 INR per month per centre (*3 centres) | 3,05,200 * 4 = 12,20,800 INR  3,30,400*3= 9,91,200 | 3,66,240*4= 14,64,960 INR  3,96,480*3= 11,89,440 | 3,20,460* 4 = 12,81,840 INR  3,46,920*3= 10,40,760 | | 71,89,000 INR |
|  | 1. Project staff Nurse (Project Nurse-II) (**5% increment in 3^rd^ year**)   @ 20,000 + 9% HRA = ~21,800 INR per month per centre (*4 centres)  @ 20,000 + 18% HRA = ~23,600 INR per month per centre (*3 centres) | 2,18,000 * 4 = 8,72,000 INR  2,36,000*3= 7,08,000 INR | 2,61,600*4 = 10,46,400 INR  2,83,200* 3= 8,49,600 INR | 2,28,900 * 4 = 9,15,600 INR  2,47,800*3= 7,43,400 INR | | 51,35,000 INR |
|  | Subtotal | 37,92,000 | 45,50,400 | 39,81,600 | | 1,23,24,000 INR |
|  | **Justification of Staff/Manpower:** Although each department has nursing staff, but the dedicated nurse is not provided by institute for antimicrobial stewardship work or project purpose. The project will need a dedicated nurse for the project to spare visit to the OT area posted and follow up of patients as the follow up is 30-days post-surgery (or 90-days if implant in situ) | | | | | |
|  | **Equipment Name** |  |  |  |  | |
|  | Desktop with Multifunctional Printer | 70,000 + 40,000 per Centre (*7) |  |  | 7,70,000 INR | |
|  | **Consumables (Recurring)** |  |  |  |  | |
|  | Printing and stationary | 50,000 per Centre (*7) |  |  | 3,50,000 INR | |
|  | **Contingency** |  |  |  |  | |
|  | Article Publication Charges (APC) for 2-3 manuscripts expected | 2,50,000 |  | 2,50,000 | 5,00,000 INR | |
|  | Miscellaneous | 2,00,000 |  |  | 2,00,000 INR | |
|  | **Travel and Meetings** |  |  |  |  | |
|  | Physical meeting of all PIs/Co-PIs: 5 (one before starting the study, three during study and one after study completion) visit to centre for training/data compilation and TA/DA to all collaborators | 1,00,000 | 3,00,000 | 1,00,000 | 5,00,000 INR | |
|  | Prescription research and prescriber skill course (including training of the JRF and ICN for data collection) at the central coordinating site | 1,00,000 | 1,00,000 | 1,00,000 | 3,00,000 INR | |
|  | Re-training sessions |  | 1,00,000 | 1,00,000 | 2,00,000 INR | |
| 6. | **Overhead charges** (3% excluding travel and meetings, equipment, consumables) | 1,27,260 | 1,36,512 | 1,26,948 | 3,90,720 INR | |
|  | **Subtotal year wise** | 56,89,260 INR | 51,86,912 INR | 46,58,548 INR |  | |
|  | **Total budget of all seven centers for 3 years** | | | | **1,55,34,720 INR** | |

# GANTT CHART

# References

1. CDC document on Surgical site infection Event (SSI). January 2023 <https://www.cdc.gov/nhsn/pdfs/pscmanual/9pscssicurrent.pdf> Last accessed on 28th March 2023.
2. WHO document on Global guidelines for the prevention of surgical site infection, second Edition. WHO Guidelines Development Group; Geneva, World Health Organization; 2018.
3. Kamat US, Fereirra AM, Kulkarni MS, Motghare DD. A prospective study of surgical site infections in a teaching hospital in Goa. Indian J Surg 2008;70:120-4
4. GlobalSurg Collaborative. Surgical site infection after gastrointestinal surgery in high-income, middle-income, and low-income countries: a prospective, international, multicentre cohort study. Lancet Infect Dis. 2018;18:516-525.
5. Anderson D, Podgorny K, Berrios-Torres SI, Bratzler DW, et al. Strategies to prevent surgical site infections in acute care hospitals:2014 update. Infect Control Hosp Epidemiol. 2014;35(6):605-627.
6. Bratzler DW, Dellinger EP, Olsen KM, et al. Clinical practice guidelines for antimicrobial prophylaxis in surgery. ASHP Report. Am J Health-Syst Pharm. 2013;70:195-283.
7. Kumar A, Rai A. Prevalence of surgical site infection in general surgery in a tertiary care centre in India. Int Surg J 2017;4:3101-6.
8. NIHR Global Research Collaborative. Reducing surgical site infections in low-income and middle-income countries (FALCON): a pragmatic, multicentre, stratified, randomised controlled trial. Lancet 2021;398:1687-99.
9. Beniwal M, Griwan MS, Marwah S et al. Incidence of surgical site infection in clean and clean-contaminated wounds: A prospective study. IJMSCR 2020;3:478-493.
10. Arora A, Bharadwaj P, Chaturvedi H, Chowbey P, Gupta S, Leaper D, et al. A review of prevention of surgical site infections in Indian hospitals based on global guidelines for the prevention of surgical site infections in Indian hospitals based on global guidelines for the prevention of surgical site infection. J Patient Saf Infect Control 2018;6:1-12.
11. Antimicrobial stewardship programmes in health-care facilities in low- and middle-income countries. A practical toolkit. Geneva: World Health Organization; 2019. Last accessed on 10^th^ May 2023.
12. <https://www.who.int/news-room/feature-stories/detail/closing-doors-stops-deadly-surgical-site-infections-in-uganda> last accessed on 10th May 2023.
13. Jamtvedt G, Young JM, Kristoffersen DT, et al.. Audit and feedback: effects on professional practice and health care outcomes. *Cochrane Database Syst Rev* 2006; 2:CD000259.
14. Khan T, Mushtaq E, Khan F et al. Decreasing the rate of surgical site infection in patients operated by cesarean section in a tertiary care hospital in India: A quality improvement initiative. Cureus 2023; 15(1):e34439.
15. Singh SK, Sengupta S, Antony R et al. Variations in antibiotic use across India: multi-centre study through Global Point Prevalence survey. J Hosp Infect 2019; 103: 280–3.
16. Saleem Z, Hassali MA, Versporten A et al. A multicenter point prevalence survey of antibiotic use in Punjab, Pakistan: findings and implications. Expert Rev Anti Infect Ther 2019; 17: 285–93.
17. Anand S, Raman D, Shetty D et al. Antibiotic prescription practices for surgical prophylaxis in India: An observational Study. Am J Trop Med Hyg 2019;101(4):919-922.
18. Cooper L, Sneddon J, Afriyie DK et al. Supporting global antimicrobial stewardship: antibiotic prophylaxis for the prevention of surgical site infection in low- and middle-income countries (LMICs): a scoping review and meta-analysis. JAC Antimicrob Resist 2020;2(3):dlaa070.
19. Mangram AJ, Horan TC, Pearson ML, et al. Guideline for Prevention of Surgical Site Infection, 1999. Infect Control Hosp Epidemiol 1999; 20:250
20. Blencowe NS, Mills N, Cook JA, et al. Standardizing and monitoring the delivery of surgical interventions in randomized clinical trials. BJS 2016; 103:1377-1384.
21. American Society of Anesthesiologists. *ASA Physical Status Classification System*. Available from: http://www.asahq.org/quality-and-practice-management/standards-guidelines- and-related-resources/asa-physical-status-classification-system. Last accessed on 20th May 2023.
22. Braun V, Clarke V. To saturate or not to saturate? Questioning data saturation as a useful concept for thematic analysis and sample-size rationales. Qualitative research in sport, exercise and health 2021;13(2):201-216.
23. Byrne D. A worked example of Braun and Clarke’s approach to reflexive thematic analysis. Qual Quant 2022; 56:1391–1412. <https://doi.org/10.1007/s11135-021-01182-y>.

# Appendix-I: Template example of the components of Multipronged interventions and CheckLIST to be checked for each patient

The intervention components can be chosen based on observed practices in the clinical setting, gap analysis, the global guidelines for prevention of surgical site infections by WHO^2^, second edition 2018 and Clinical practice guidelines for antimicrobial prophylaxis in surgery which were recommended with “Strong” recommendations and “Moderate to Good” quality of evidence and easy to implement in the setting.

**Table-2:** Template intervention package (not necessarily the same interventions mentioned below)

| **Components of multipronged Intervention** | **Steps of the Intervention** | **Type of standardization** | **Limits of standardization** |
| --- | --- | --- | --- |
| Interventions for surgical infection prevention: four system interventions | Delivery of intravenous antimicrobial prophylaxis within 1 hour before incision (2 hour for vancomycin and fluoroquinolones) | Mandatory | Boundaries (Flexibility in choosing antimicrobial by the surgeon based on the type of surgery, patient factors, clinical practice and guidance documents) |
|  | Redosing of antimicrobial if interval >2 half-lives or if blood loss >1500 ml | Optional | Boundaries (Flexibility based on the duration of the surgery and type of the antimicrobial agent chosen for surgical prophylaxis) |
|  | Use of antimicrobial prophylactic agent consistent with published guidelines (Global guidelines for prevention of surgical site infections by WHO^2^, second edition 2018 and Clinical practice guidelines for antimicrobial prophylaxis in surgery^10^) | Mandatory | Boundaries (Flexibility in choosing antimicrobial by the surgeon based on the type of surgery, patient factors, clinical practice and guidance documents) |
|  | Discontinuation of prophylactic antimicrobial agent within 24 hours of surgery (within 48 hours for cardiothoracic procedures in adult patients): Post operative day- 1(POD-1) review | Mandatory | Flexibility allowed till 24 hours postoperative |
| Interventions for surgical care improvement: five behavioral interventions | Proper hair removal: No removal (if not hampering surgery) or use of clippers/depilator method. | Optional | Boundaries (Flexibility in choosing no removal or clippers or depilators) |
|  | Use of razors for hair removal | Prohibited (Considered inappropriate if razors used for hair removal) | Exactly (Not flexible) |
|  | Use of alcohol chlorhexidine based pre-operative skin preparation if no contra-indication exists and aseptic precautions including hand hygiene. | Optional (Based on availability and patient factors, alcohol chlorhexidine based or Povidone iodine-based solution can be used for skin preparation with proper drying) | Flexible based on availability and surgeon choice |
|  | Controlled blood glucose during immediate post-operative period. | Optional | Boundaries (levels blood sugar to be mentioned as controlled varies as per patient characteristics) |
|  | Maintenance of perioperative normothermia (≥35.5°C) in surgical patients | Optional (maintained if duration of anesthesia >60 minutes) | Exactly (Patients where therapeutic hypothermia needed will be excluded from the study) |
|  | Minimizing the door opening of the operating room | Optional | Flexible |

**Table-3: Template Checklist to be checked for each patient based on the components of the multipronged interventions co-developed by the stakeholders.**

| S.NO | Interventions |  | Intervention followed or not (Yes or No) |
| --- | --- | --- | --- |
| 1 | Delivery of intravenous antimicrobial prophylaxis within 1 hour before incision (2 hr for vancomycin and fluoroquinolones) | Time of antimicrobial prophylaxis administration…………..  Time of start of surgery…………………. |  |
| 2 | Redosing of antimicrobial if interval >2 half-lives or if blood loss >1500 ml | Redosing required or not …….  Duration of surgery ………… |  |
| 3 | Use of antimicrobial prophylactic agent consistent with published guidelines | Name of antimicrobial given as surgical prophylaxis …………………………. |  |
| 4 | Discontinuation of prophylactic antimicrobial agent within 24 hours of surgery (within 48 hours for cardiothoracic procedures in adult patients) | Time of stoppage of surgery……………  Time and date of last dose of antimicrobial prophylaxis …………………………….. |  |
| 5 | Proper hair removal: No removal (if not hampering surgery) or use of clippers/depilator method. Use of razors to be considered inappropriate | Hair removed or not ………  If yes, method of hair removal …………………………… |  |
| 6 | Use of alcohol chlorhexidine based pre-operative skin preparation if no contra-indication exists and aseptic precautions including hand hygiene. | Agent used for skin preparation……………….. |  |
| 7 | Controlled blood glucose during immediate post-operative period. | Blood sugar levels 6 hourly in postoperative period …………  …………………………… |  |
| 8 | Maintenance of perioperative normothermia (≥35.5°C) in surgical patients who have anesthesia >60 minutes | Temperature in intra-operative period ………….. |  |
| 9 | POD-1 review of antimicrobials. | Any advice given: ……………………………….  Advice followed or not? …………………………….. |  |
| 10 | SSI surveillance as per CDC/NHSN definition of SSI. | SSI surveillance done as per definition mentioned in Appendix-II …………….. |  |

# Appendix-II: who SURGICAL SITE INFECTION SURVEILLANCE POST-OPERATIVE DATA COLLECTION FORM

# Appendix-IiI: DIAGNOSIS OF SURGICAL SITE INFECTION AS PER CDC

| **Superficial incisional SSI :** Must meet the following criteria:  Date of event occurs within 30 days following the operative procedure (where day 1 = the procedure date) **AND** involves only skin and subcutaneous tissue of the incision  **AND**  patient has at least ***one*** of the following:   1. purulent drainage from the superficial incision. 2. organism(s) identified from an aseptically-obtained specimen from the superficial incision or subcutaneous tissue by a culture or non- culture based microbiologic testing method which is performed for purposes of clinical diagnosis or treatment (for example, not Active Surveillance Culture/Testing [ASC/AST]). 3. a superficial incision that is deliberately opened by a surgeon, physician* or physician designee and culture or non-culture based testing of the superficial incision or subcutaneous tissue is not performed **AND** patient has at least one of the following signs or symptoms: localized pain or tenderness; localized swelling; erythema; or heat. 4. diagnosis of a superficial incisional SSI by a physician. |
| --- |
| **Deep incisional SSI**  Must meet the following criteria:  Date of event occurs within 30 or 90 days following the operative procedure (where day 1 = the procedure date) **AND** involves deep soft tissues of the incision (for example, fascial and muscle layers) **AND** patient has at least ***one*** of the following:   1. purulent drainage from the deep incision. 2. a deep incision that is deliberately opened or aspirated by a surgeon, physician or spontaneously dehisces   **AND**  organism(s) identified from the deep soft tissues of the incision by a culture or non-culture based microbiologic testing method which is performed for purposes of clinical diagnosis or treatment (for example, not Active Surveillance Culture/Testing [ASC/AST]) or culture or non- culture based microbiologic testing method is not performed. A culture or non-culture based test from the deep soft tissues of the incision that has a negative finding does not meet this criterion.  **AND**  patient has at least ***one*** of the following signs or symptoms: fever (>38°C); localized pain or tenderness.   1. an abscess or other evidence of infection involving the deep incision detected on gross anatomical exam, histopathologic exam, or imaging test. |

# Appendix-IV: SURGICAL WOUND CLASSIFICATION AS PER CDC

| Surgical wound classification as per CDC definition^2,19^ | |
| --- | --- |
| Class I/ Clean | An uninfected operative wound in which no inflammation is encountered and the respiratory, alimentary, genital, or uninfected urinary tract is not entered.  In addition, clean wounds are primarily closed and, if necessary, drained with closed drainage.  Operative incisional wounds that follow nonpenetrating (blunt) trauma should be included in this category if they meet the criteria. |
| Class II/ Clean-Contaminated | An operative wound in which the respiratory, alimentary, genital, or urinary tracts are entered under controlled conditions and without unusual contamination.  Specifically, operations involving the biliary tract, appendix, vagina, and oropharynx are included in this category, provided no evidence of infection or major break in technique is encountered. |
| Class III/ Contaminated | Open, fresh, accidental wounds.  In addition, operations with major breaks in sterile technique (e.g., open cardiac massage) or gross spillage from the gastrointestinal tract, and incisions in which acute, non-purulent inflammation is encountered are included in this category. |
| Class IV/ Dirty-Infected | Old traumatic wounds with retained devitalized tissue and those that involve existing clinical infection or perforated viscera.  This definition suggests that the organisms causing postoperative infection were present in the operative field before the operation |

*Mangram AJ, Horan TC, Pearson ML, et al. Guideline for Prevention of Surgical Site Infection, 1999. Infect Control Hosp Epidemiol 1999; 20:250. Available at:*[*https://stacks.cdc.gov/view/cdc/7160*](https://stacks.cdc.gov/view/cdc/7160)*(Last Accessed on April 20, 2023).*

# Appendix-V: American society of anesthesiologist (ASA) physical status classification AS PER CDC

Each patient will be assessed by the anesthesiologist of the patient’s preoperative physical condition using the American Society of Anesthesiologists’ (ASA) Physical status classification system^2,20^. Patients will be assigned an ASA score 1-6 at the time of surgery. Patients with an ASA score of 1-5 will be eligible for SSI surveillance. Patients that will be assigned an ASA score of 6 (declared brain -dead patient whose organs are being removed for donor purposed) will not be eligible for SSI surveillance and will be excluded from the study.

| **ASA PS Classification** | **Definition** | **Adult examples, including but not limited to:** |
| --- | --- | --- |
| ASA-1 | A normal healthy patient | Healthy, non-smoking, no or minimal alcohol use |
| ASA-II | A patient with mild systemic disease | Mild disease only without substantive functional limitations. Current smoker, social alcohol drinker, pregnancy^*^, obesity (30<BMI<40), well controlled DM/HTN, mild lung disease |
| ASA-III | A patient with severe systemic disease | Substantive functional limitations; One or more moderate to severe diseases. Poorly controlled DM or HTN, COPD, morbid obesity (BMI ≥40), active hepatitis, alcohol dependence or abuse, implanted pacemaker, moderate reduction of ejection fraction, ESRD undergoing regularly scheduled dialysis, history (>3 months) of MI, CVA, TIA, or CAD/stents. |
| ASA-IV | A patient with severe systemic disease that is a constant threat to life | Recent (<3 months) MI, CVA, TIA or CAD/stents, ongoing cardiac ischemia or severe valve dysfunction, sever reduction of ejection fraction, shock, sepsis, DIC, ARD or ESRD not undergoing regularly scheduled dialysis. |
| ASA-V | A moribund patient who is not expected to survive without the operation | Ruptured abdominal/thoracic aneurysm, massive trauma, intracranial bleed with mass effect, ischemic bowel in the face of significant cardiac pathology or multiple organ/system dysfunction |
| ASA-VI | A declared brain-dead patient whose organs are being removed for donor purposes. |  |
| * Although pregnancy is not a disease, the parturient’ s physiologic state is significantly altered from when the woman is not pregnant, hence the assignment of ASA 2 for a woman with uncomplicated pregnancy. | | |
